# Hypertension is a genetic condition—a quantum dilemma

**DOI:** 10.1038/s41371-024-00898-2

**Published:** 2024-02-20

**Authors:** Rebecca Hanna

**Affiliations:** NHS Scotland, Research Fellow at University of Glasgow (School of Cardiovascular & Metabolic Health), Glasgow, Scotland UK

**Keywords:** Hypertension, Diagnosis, Genetics research, Risk factors, Prognosis

The British Irish Hypertension Society (BIHS) 2023 Sir Stanley Peart Essay Competition posed a challenging topic: ‘Hypertension is a genetic condition?’ The presence of a question mark in the topic immediately conveys the uncertainty surrounding the genetic underpinnings of hypertension. In this prize-winning essay, I delve into the causes, complications and management of hypertension from a patient perspective intertwining the latest genetic evidence with clinical, pathophysiological and epidemiologic insights while also considering the impact of cognitive biases. This essay illuminates the complex tapestry of hypertension’s origins, emphasising the intricate interplay between genetics, environment, clinical practice, and patient beliefs which collectively contribute to the significant public health burden posed by hypertension.

Amidst the towering dunes and unfamiliar terrain of the left cerebral hemisphere, a mysterious tale unfolds inside the body of a brilliant quantum physicist. Our protagonist, a determined macrophage, finds itself entangled in a thrilling investigation of a mystery: a complete thrombosis of the middle cerebral artery, resulting in a devastating stroke. Little did he know that this journey would lead him to probe the complex basis of hypertension and question whether it was truly a genetic condition.

‘Hurry macrophage! We’re needed at penumbra!’ cries a frantic neutrophil, thus setting the macrophage on its journey of discovery. As the macrophage navigates the cerebral landscape, it passes concerned and fearful neurons until it reaches the penumbra where neurons lie gasping for oxygen. Beyond the penumbra, it sees the infarct core strewn with lifeless neurons.

‘They are blaming hypertension for this, but they are wrong. Our own genes are responsible…’ gasps a nearby penumbral neuron. Curiosity piqued, the macrophage delves into its immunological and evolutionary memory [1], embedded in its genome and epigenome, and only finds that thrombosis blocking the artery is the likely cause of the stroke. About hypertension, the information available is that it damages blood vessel walls where in due course some foamy comrades of the macrophage take up residence. It cannot find anything about genes, but then it realises that it may only possess information pertinent to its role as a simple scavenging macrophage. The neurons pointing to genes as the culprit makes the macrophage determined to find more answers.

‘Could you provide more details so that I may better assist you?’ the macrophage asks the dying neuron. The macrophage learns that the quantum physicist was recently diagnosed with hypertension and had been engrossed in finding out the reasons for developing the condition. The neuron’s fading voice directs the macrophage to travel to Broca’s area to find out what the physicist was discussing recently.

## A duel of ideas

The Broca neurons enlighten the macrophage about the quantum physicist’s last conversations. The macrophage learns that just before his stroke, the quantum physicist was engaged in discussions with various people about the causation of hypertension. The macrophage stumbles upon the remnants of a heated debate between two theories. Platt’s proposition of a genetic basis for hypertension clashed with Pickering’s data-driven perspective of multifactorial causation, in other words the quarrel was whether hypertension was due to single genetic mutations with large effects on blood pressure (Platt), or if hypertension was the net effect of hundreds of genetic variations and environmental factors (Pickering) [2]. Other arguments were more in favour of an environmental or lifestyle basis—as far back as 2600 BC, correlations were observed between salt intake, high blood pressure, stroke, and kidney disease [3]. ‘Look, macrophage,’ a Broca neuron speaks gently. ‘Go to the hippocampus. We maintain records of everything there—the memory of everything read, heard, and understood. I believe you’ll find some of the answers you seek there.’

## A quantum lens on the puzzle

Venturing into the hippocampus, the macrophage encounters a world of quantum mysteries interwoven with new insights about hypertension. Brushing against neurons evokes flashes of electrical sparks within complex synaptic networks allowing the macrophage to absorb the stored information. Unsurprisingly, most of the data revolves around wave-particle duality, quantum entanglement, and the nature of reality [4]. However, amidst this quantum realm, the macrophage stumbles upon some recently formed neuronal networks containing information about hypertension and the conundrum over genetic or environmental causation. ‘Haha, how typical of a quantum physicist to invoke Schrodinger’s cat to explain the duality of hypertension’s origins,’ [5] the macrophage muses. ‘How absurd! Even I understand that quantum phenomena manifest only at the subatomic level and bear no relevance to explaining macroscopic and physiological processes [6]. I proudly identify as a macrophage, not some elusive wave,’ the macrophage declares, slightly exasperated by the far-fetched association.

## A riddle, wrapped in a mystery, shrouded in an enigma

The macrophage acknowledges various evolutionary hypotheses of hypertension indicating a genetic basis—is hypertension a maladaptive response of genes that were selected for salt retention when life moved from salt-rich oceans to salt-poor land? More recently, is the increased risk of salt-sensitive hypertension among African Americans related to enrichment of salt conserving gene variants amongst the survivors of the brutal transatlantic slave transports? [7, 8] Curiously, the maladaptive gene theory stumbles with data showing lower prevalence of hypertension among rural populations and in unacculturated societies like the Yanomami Indians [9]. Furthermore, migration of rural individuals to urban areas results in an increased incidence of hypertension [10]. All these point to an environmental cause. There were other inconsistencies: the peculiar cases of Gitelman Syndrome (affected individuals would crave salt but maintained low blood pressure despite high salt intake) and Glucocorticoid-remediable aldosteronism (high blood pressure paradoxically resolved with corticosteroid treatment) clearly show the role of genes with mutations identified in *SLC12A1* gene for the former and a chimeric translocation between *CYP11B1* and *CYP11B2* genes for the latter [3]. The INTERSALT study [11] demonstrated a causal effect of sodium intake on blood pressure, but salt-sensitivity is a normally distributed phenotype implying multifactorial influences—both genetic and environmental.

The fraction of population blood pressure variation explained by genetic variation is quantified as heritability and the estimated heritability of blood pressure is modest at around 15–40% for clinic blood pressure but higher for ambulatory blood pressure (50–69%) [12]. This formed the basis for the highly successful drive to discover the polygenic architecture of blood pressure and hypertension. The genomic era over the last 15 years, specifically through a plethora of genome wide association studies (GWAS) identified over 1500 single nucleotide polymorphisms (SNPs) associated with blood pressure and hypertension [12]. Yet it also carried a sense of disappointment as researchers struggled to translate genetic signals into clinical applications. All the GWAS SNPs together explained only approximately 27% of the estimated blood pressure heritability and 5.7% of the phenotypic variance of SBP [12]. Combining all the SNPs into a polygenic score to predict hypertension has not yet shown clinical utility. GWAS in general have shown potential to discover novel pathways, but clinical translation is challenging. The deflated expectations from GWAS for rapidly using genomics to understand and treat hypertension echoed the similar over-expectations following the first sequencing of the human genome in 2001 [13]. It dawns on the macrophage that the experimental data on blood pressure and hypertension seemed to suggest either an environmental or a genetic causal mechanism, depending on who conducted the studies, indicating a form of confirmation bias. ‘Perhaps, like quantum particles, hypertension exists in a state of causal superposition, where seemingly incongruous factors intertwine to shape its manifestation,’ it mused. The macrophage’s early criticism of the quantum physicist considerably waned, sympathising with the physicist likening the whirlwind of contradictory information to the complex interplay of subatomic particles (or waves) entangled in a quantum dance.

## The renal imperative

Undeterred, the macrophage ventures to the kidneys, central to unravelling the mystery. Engulfed by a cacophony of pumps and engines, the macrophage witnesses the intricate dance of sodium and water balance orchestrated by pumps working hard with teutonic efficiency and discipline. The pumps confirm that their vital role in regulating sodium doesn’t involve genes directly, and genetic mutations leading to monogenic syndromes are rare and usually severely disrupt nephron function. A sodium-hydrogen pump during a brief respite pitches in, ‘We work physiologically and linking our sodium homoeostasis imperative with essential hypertension is best explained by Guyton’s pressure-natriuresis theory [14] which suggests that when blood pressure rises, we automatically respond by increasing the excretion of sodium and water thereby reducing blood pressure back to normal. However, prolonged high salt intake can reset this equilibrium point, leading to sustained salt retention and consequently hypertension. This is a long-term effect of high salt intake resulting in maladaptation of our fine-tuned mechanisms. So far, no genes have been discovered causing pressure natriuresis. It is all hard-working nephrons toiling 24/7’. Further down the nephron, an epithelial sodium channel hails the macrophage. It says, ‘occasionally some *schwachkopf* eats a lot of licorice and that messes with an enzyme called 11-HSDB2 resulting in unmasking cortisol’s mineralocorticoid effect and thence sodium retention and hypertension—a condition called Apparent Mineralocorticoid Excess [3]. So, dietary factors other than salt intake can mimic excess salt intake through interference with gene function resulting in high blood pressure—this is an example of an indirect effect of genes.’

## Seeing through a glass darkly

The macrophage decides to skip a visit to the adrenals to piece together all the information acquired in its investigation thus far. In summary, hypertension entails high blood pressure acting on blood vessels and, if left untreated, can lead to severe complications such as strokes and heart attacks. It vividly recalls passing by an area of thrombosis in a grotty-looking artery not long ago and specifically noticing the presence of lumpy yellowish plaques (his foamy friends), especially near arterial junctions. The macrophage’s platelet comrades have been increasingly discontented of late, burdened by the escalating workload over the past few years. The macrophage did not know the reason for this, but now realises that this must be because of blood vessel wall damage caused by hypertension. Likewise, the macrophage itself has been increasingly called upon, tasked with cleaning up various arterial issues. The fog of uncertainty begins to lift as the macrophage connects the dots between hypertension and the ongoing cerebral catastrophe.

## A trail of missed opportunities

As the macrophage approaches the penumbra once more, it has come full circle in its investigation. It finally understands the intricate connections between genetics, hypertension, and the stroke that had befallen the quantum physicist. In a moment of solemn realisation, the macrophage stands amidst the aftermath of the cerebral catastrophe. The truth is crystal clear, and it is time to deliver its findings. The genetic influence on blood pressure is tempered by a multitude of factors, resulting in the final resultant blood pressure phenotype. Thus, debating whether hypertension is a genetic disease invokes a false dichotomy and a trip down a rabbit hole rather than focusing on clinical reality. ‘The hypertension that led to this stroke is not a mere illusion or a matter of quantum uncertainty,” it declares, ‘but a reality that demands attention and proper management [15].” The quantum physicist’s relentless pursuit of knowledge had been admirable, but his fervour blinded him to the immediate danger. The quantum physicist’s stroke was a tragic consequence of overlooking the significance of immediate medical intervention and a stark reminder that even the brightest minds can be vulnerable to the silent dangers of hypertension. In the realm of medicine, actions based on the knowledge available are just as vital as seeking new answers about the ultimate truth of causality [15].

## Epilogue: seeking balance

**Fig. 1:**
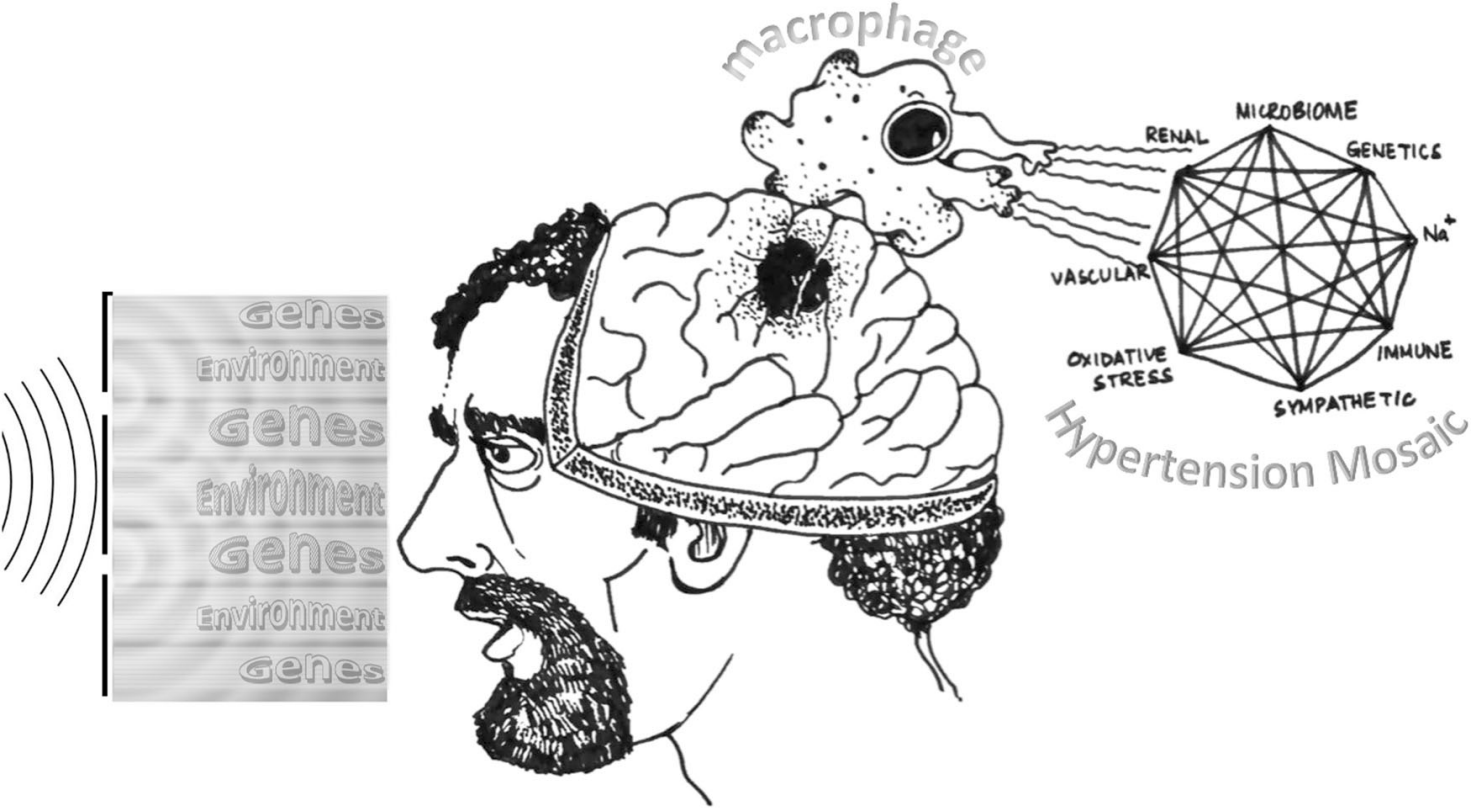
The Dunning-Kruger effect and hypertension. The figure illustrates how a cognitive bias, known as the Dunning-Kruger effect, can lead to a mismatch between one’s perception and reality of hypertension and its consequences. The left panel shows a quantum physicist who suffers from high blood pressure, trying to explain his condition using his expertise in wave-particle duality. This results in him ignoring the actual causes of hypertension, such as smoking, obesity, salt intake, and alcohol consumption, and delaying appropriate treatment. The middle panel shows the same physicist having a stroke. The right panel shows a macrophage investigating the reason for the stroke and discovers the complex and multifactorial nature of hypertension. The macrophage also reveals the dangers of the Dunning-Kruger effect, which can make people overestimate their own knowledge and competence and underestimate the expertise of others. The macrophage warns that faulty reasoning, inaccurate judgments, and erroneous conclusions can have detrimental effects on one’s health and well-being. In this essay, the macrophage explores the causes, mechanisms, and prevention of hypertension, and the role of critical thinking and evidence-based decision-making in avoiding the Dunning-Kruger effect. Illustration by Dr. Kushal K Choudhuri.

The macrophage recognises that the causation of hypertension (genetic or environmental or lifestyle) and quantum physics (measurement problem) may share certain conceptual parallels, but their application must remain distinct. While the mysteries of quantum mechanics may continue to elude understanding, the urgency of early diagnosis and prompt medical treatment of hypertension requires practical and evidence-based actions. In the end, the journey through the quantum physicist’s body taught the macrophage that medicine and science demand a delicate balance between exploration and application. As the body’s sentinel, the macrophage resolves to continue its mission, ever vigilant in maintaining harmony within the complex microcosm of the human body (Fig. 1).
